# How Is Lebanon’s Progress Towards Measles Elimination? Review of Surveillance Performance Indicators, 2013–2024

**DOI:** 10.3390/epidemiologia7030069

**Published:** 2026-05-14

**Authors:** Lina Chaito, Pawel Stefanoff, Hawraa Sweidan, May Younes, Mona Albuaini, Nada Ghosn

**Affiliations:** 1 Epidemiological Surveillance Program, Ministry of Public Health, Beirut 0127, Lebanon; hawraa.sweidan@gmail.com (H.S.); mayiyouness@gmail.com (M.Y.);; 2 Mediterranean and Black Sea Programme in Intervention Epidemiology Training (MediPIET), European Centre for Disease Prevention and Control (ECDC), 171 83 Stockholm, Sweden; 3 National Measles Laboratory (NML), Rafic Hariri University Hospital (RHUH), Beirut 0127, Lebanon

**Keywords:** measles, surveillance, indicators, elimination, Lebanon

## Abstract

Background: Lebanon adopted the World Health Organization (WHO)’s regional strategic plan (2012–2020) to achieve measles elimination. We aimed to analyze WHO measles surveillance performance indicators to identify areas for improvement. Methods: We reviewed suspected measles and rubella cases notified to the national epidemiological surveillance program between January 2013 and December 2024. A suspected case was defined as any patient with a febrile maculopapular rash or considered clinically compatible by physicians. We assessed notification rates of discarded non-measles/rubella suspected cases, timeliness of investigations within 48 h, completeness of case investigations (demographic and vaccination data), and proportion of cases tested for measles/rubella. Mean proportions and standard deviations were calculated across years and provinces. Results: A total of 6581 suspected cases were reported, predominantly from hospitals (66%). Outbreaks occurred in 2013 (*n* = 1760), 2018–2019 (*n* = 1984) and 2023–2024 (*n* = 346). Outside outbreak years, the notification rate ranged between 0.7 and 1.8 per 100,000 population. Timely investigation was achieved in 72% (±30%) of cases, while adequate investigation reached 52% (±19%). Laboratory testing was performed in 62% (±16%) of cases. Conclusions: Surveillance in Lebanon showed suboptimal sensitivity, a high proportion of hospitalized cases, and variability in completeness of epidemiological and laboratory investigations. Strengthening outpatient reporting and continuous training of healthcare professionals are essential to improve surveillance performance.

## 1. Introduction

The measles initiative was launched in 2001 by a global partnership between the World Health Organization (WHO), United Nations International Children’s Emergency Fund (UNICEF) and other partners aiming to reduce mortality and morbidity caused by the highly contagious measles virus. The initiative contributed significantly to global public health, achieving an 83% reduction in measles deaths between 2000 and 2021. As part of its broader goals, the initiative also promoted the integration of rubella control and elimination efforts, eventually leading to the formation of the Measles and Rubella Initiative (M&RI) in 2012 [1,2].

In 2015, all 22 countries and areas within the World Health Organization’s Eastern Mediterranean Region (EMR) committed to eliminating measles by 2020 as part of the Vaccine Action Plan for this region. The main components of this action plan were to increase population immunity through vaccinating at least 95% children with two doses of measles-containing vaccine and efficient measles and rubella surveillance. Despite all efforts, the elimination goal was not met in most of the EMR countries due to several challenges [3].

Lebanon adopted the strategic plan of the EMR towards achieving measles elimination. However, indigenous measles virus transmission remains a public health concern in Lebanon and national outbreaks occur every 4–5 years [4]. In 2001, the Epidemiological Surveillance Unit (ESU) of the Ministry of Public Health (MOPH) implemented measles surveillance with epidemiological and laboratory investigation. Measles surveillance was integrated with acquired rubella surveillance, due to similar clinical picture and vaccines. The program was monitored through a set of indicators assessing surveillance sensitivity and performance. Surveillance, monitoring and evaluation are essential tasks at all levels to improve performance and identify and address problems [5].

We aimed to review surveillance performance indicators in Lebanon to provide recommendations for improvement.

## 2. Materials and Methods

We reviewed suspected cases of measles, notified to the national epidemiological surveillance program between January 2013 and December 2024. Health professionals were mandated to notify within 24 h each case with symptoms compatible with measles to ESU by completing an individual case reporting form. Until 2022, notification forms were primarily submitted via fax or WhatsApp (V2.20 to v2.23). Starting in 2023, case-based reporting transitioned to the online District Health Information System 2 (DHIS2) platform. Upon reception, epidemiologists at ESU investigated each suspected case and ensured that collected specimens were transported to the national measles laboratory (NML). The specimens most commonly collected were serum or oral fluid. Collected specimens across all regions, were transported from reporting sites across Lebanon to the central level of the Epidemiological Surveillance Unit (ESU), where they were checked and labeled before being sent to theNML. Specimens were considered adequate for measles testing if they met predefined criteria regarding timing of collection, sample quantity, and storage conditions. The acceptable interval between specimen collection and laboratory analysis varied according to the specimen type and the specific test performed. In addition, specimens were required to have sufficient volume for the requested analyses and to be properly collected, labeled, and transported under appropriate conditions, maintained at 4–8 °C, to preserve sample integrity.

**Figure 1:**
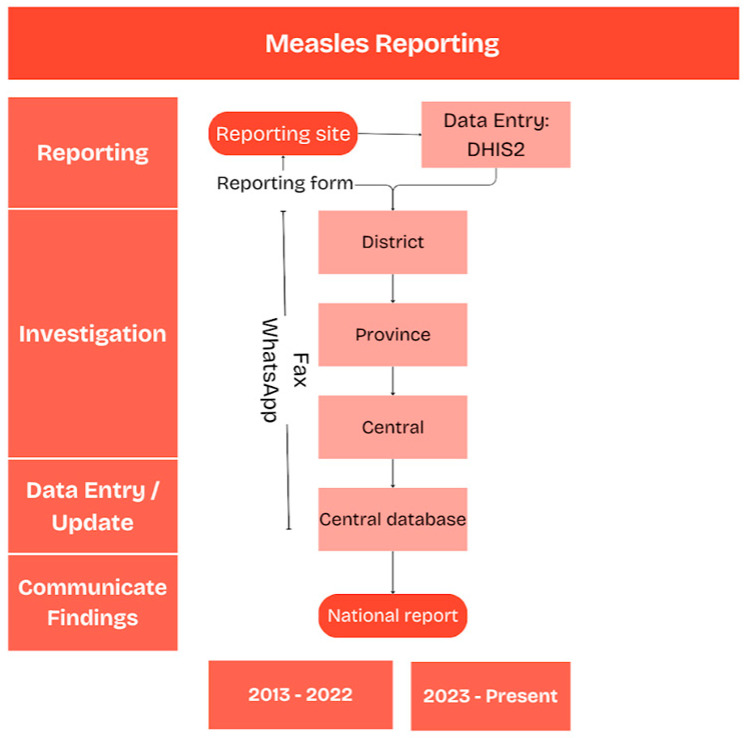
Flow chat of surveillance data across different levels, Lebanon, 2013–2024.

All suspected measles cases were routinely tested for both measles and Rubella. Laboratory results were communicated back by fax or shared Google document. A detailed report summarizing main findings was communicated to the Expanded Immunization Program (EPI) at MOPH and other relevant stakeholders (Figure 1). A suspected measles case was defined as a person with fever >38 °C and maculo-papular rash or any patient for whom the physician suspected measles. A clinically compatible case was defined as a suspected measles case without laboratory confirmation (or with equivocal results) and without epidemiological linkage to a confirmed case. A laboratory confirmed case was defined as a suspected case confirmed as measles by detecting measles-specific IgM antibodies or measles virus RNA in collected samples. An epidemiologically linked case was defined as a suspected case that was in close contact with a laboratory-confirmed measles case within the last 28 days before the onset of rash. Following the completion of epidemiological and laboratory investigations, the ESU was responsible for the final classification of cases. We calculated measles surveillance performance indicators based on the WHO surveillance performance protocol (Table 1) [6]. We calculated mean proportions and standard deviations to summarize measles surveillance performance indicators per year and per province. To further analyze one of the key surveillance performance indicators, adequacy of case investigation, we assessed the completeness of relevant variables by calculating the number and proportion of missing data for each targeted field.

Ethical approval was not required as the measles surveillance is within the essential public health functions of the Ministry of Public Health.

## 3. Results

### 3.1. Epidemiology of Measles in Lebanon

Between January 2013 and December 2024, a total of 6581 suspected measles cases were reported to the ESU. Of these, 2514 (38%) were confirmed by laboratory testing, 2076 (32%) were classified as clinically compatible, 154 (2%) were identified as epidemiologically linked, 146 (2%) were classified as rubella cases, and 1691 (26%) were discarded after testing negative for both measles and rubella. The average incidence over the study period was 67.5 (±97.4) per 1,000,000 population for measles and 2.21 (±1.61) per 1,000,000 population for rubella.

**Figure 2:**
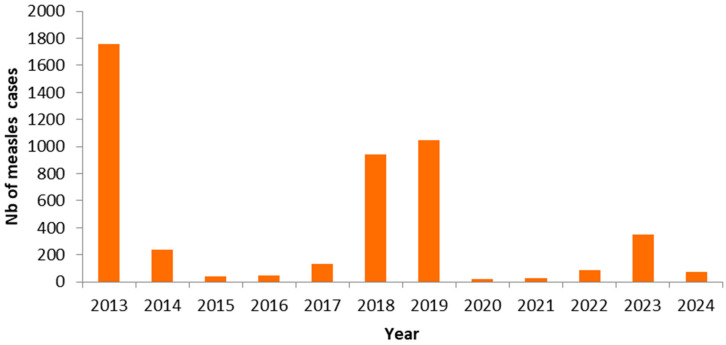
Reported measles by year of notification, Lebanon, 2013–2024.

Three national measles outbreaks occurred in Lebanon during the study period. The first, in 2013, resulted in 1760 reported cases. This was followed by two subsequent waves in 2018–2019, during which 1984 cases were reported. The most recent outbreak occurred in 2023–2024, with 420 reported cases. We presented suspected, laboratory-confirmed, and epidemiologically linked measles cases reported to the surveillance system during the study period in Figure 2. Across all outbreaks, the most affected age groups were children aged 1–4 years (43%) and 5–10 years (22%), with nearly half of reported cases unvaccinated (46%), pointing to persistent immunity gaps in the population.

### 3.2. Assessment of Measles Surveillance Performance Indicators

Table 2 and Table 3 summarize the performance of selected surveillance indicators by year (2013–2024) and by Lebanese provinces. Overall, substantial variability was observed across indicators, with notable differences between outbreak and non-outbreak periods, as well as across geographic regions.

### 3.3. Notification of Suspected Measles Cases

The annual notification rate of suspected measles cases per 100,000 population showed variation across the study years. During outbreak years, the rate was 4.2 in 2013, 3.8 in 2018, 5.5 in 2019, and 6.2 in 2023. In non-outbreak years, the rate ranged from 0.7 to 1.8. Between 2013 and 2024, the provinces with the highest average reporting rates were Baalbeck-Hermel (10), Akkar (3.4), and Bekaa (2.8). In 2013–2022, most suspected cases were reported among hospital inpatients (66%), followed by outpatients visiting medical centers (14%). Less frequently, cases were notified from refugee camps and other vulnerable areas (10%), medical mobile units (6%), private clinics (2%), laboratories (2%), and schools (3%). In 2023–2024, suspected measles cases were mainly reported by medical centers (34%), field investigations (32%), followed by hospitals (19%), laboratories (10%) and schools (5%).

### 3.4. Investigation of Suspected Measles Cases

On average, 72% (±30%) of suspected measles cases were investigated within 48 h of notification, with annual proportions ranging from 16% to 100%. In contrast, the proportion of cases that were adequately investigated averaged 52%, ranging from 25% to 83%.

Overall, data completeness was high for most variables, with over 97% completeness for key demographic and surveillance indicators such as sex, age, province, and dates of rash onset and investigation. In contrast, vaccination-related variables showed substantially lower completeness, with vaccination status missing in 20% of cases and dates of first and second measles-containing vaccine doses missing in 51% and 66% of cases, respectively.

### 3.5. Laboratory Testing of Suspected Measles Cases

On average, 62% (±16%) of suspected measles cases were laboratory tested for measles and rubella, with annual proportions ranging from 28% to 79%. The highest testing rates were recorded in the governorates of Beirut (71%), South (70%), and Nabatieh (78%).

### 3.6. Adequacy of Collected Specimens

The proportion of specimens from suspect cases, received in adequate condition, averaged 98% (±1%), with annual values ranging from 96% to 100%.

### 3.7. Transportation of Collected Specimens

The proportion of specimens transported within five days of collection averaged 30% (±23%), with annual values ranging from 0% to 70%. This indicator declined markedly after 2018 (19%) and dropped to 0% during the COVID-19 pandemic in 2020–2021. The lowest proportions of timely transported specimens were observed in provinces furthest from Beirut, including Baalbeck-Hermel (17%), North (19%), and Akkar (27%).

### 3.8. Reporting of Laboratory Results

The proportion of laboratory results reported within four days of specimen reception at the national referral laboratory averaged 55% (±33%), with annual values ranging from 10% to 100%. A significant decline in this indicator was observed in 2018 (25%), 2019 (15%), 2020 (10%), and 2024 (19%) during the economic crisis and the COVID-19 pandemic.

## 4. Discussion

Our analysis of measles surveillance performance in Lebanon highlighted both strengths and limitations within the measles surveillance system. Several factors had an impact on its performance. Surveillance sensitivity appears to be affected by the occurrence of outbreaks and the implementation of electronic reporting. The annual notification rate for discarded measles cases per 100,000 population reached the recommended target only during outbreak years and in 2024 and remained below the recommended target during non-outbreak years. During outbreaks, increased awareness among healthcare providers, intensified surveillance activities, and active case finding likely contribute to higher detection and reporting of suspected cases. In contrast, during non-outbreak periods, surveillance relies primarily on passive reporting, which may lead to under-detection of cases. In addition, since 2022, we have noticed a higher rate of discarded measles cases, likely due to the transition to online reporting via DHIS2, which appears to have improved data reporting efficiency and engagement with the surveillance system. The predominance of suspected cases among inpatients suggests a gap in detection of mild cases and thus undetected community transmission. Another point of interest was the absence of regular information about vaccination coverage to guide surveillance enhancement activities. The vaccine coverage generated by the administrative information system covered only the vaccination activity in the public sector. Nevertheless, the cluster survey to measure the vaccination coverage including both public and private sectors was conducted every 4–5 years [7]. A similar situation of under-reporting has been observed in other WHO EMRO countries. For instance, Iraq and Somalia have consistently reported discarded case rates below target levels, reflecting low surveillance sensitivity. Likewise, Syria and Sudan have reported significantly suboptimal rates, largely due to conflict-related disruptions and weakened health infrastructure [8,9].

For the timeliness of investigation, around 77% of suspected cases were investigated within 48 h of rash onset, except during the period 2022–2024. Several factors may explain the decline in timely investigation including turnover of staff at ESU and in health facilities, occurrence of the cholera outbreak 2022–2023 that was considered a national emergency with more than 8000 cases, and the armed conflict 2023–2024 with large internal population displacement [10]. However, throughout the study period, the adequacy of investigations remained below the target level. This was primarily due to incomplete or missing vaccination dates. Caregivers of the investigated children often reported having lost their children’s vaccination cards or were uncertain whether their children had been vaccinated before. Our results were concordant with investigations from Iraq, Sudan and Yemen indicating similar challenges with documentation of vaccination status and caregivers recall [8,9,11]. On the other hand, the MOPH initiated an application to register childhood vaccination status, including dates of vaccination. Yet, this database did not cover all the children and was not accessible to the ESU staff.

Another major concern was laboratory investigation of reported measles cases. Outside the pandemic period, the proportion of laboratory-confirmed cases ranged between 58% and 79%, but it dropped sharply to 28–52% during the COVID-19 pandemic. This operational gap was more pronounced in primary healthcare settings where patient turnover was high and the integration of surveillance protocols into clinical practices was considered weak with certain disparity across different regions [12]. In addition, the testing of oral fluid specimens was discontinued in 2020 because the needed laboratory reagents were not procured. Adequate specimen collection can enhance the specificity of surveillance data and prevent an underestimation of measles incidence [13]. In addition, laboratory-confirmed cases provided the basis for outbreak detection, confirmation and response [3]. Moreover, efficient collection of specimens enables genotyping to identify circulating strains and document the interruption of endemic transmission [6,13].

The national reference laboratory received adequate specimens during the investigation period. The MOPH provided all ESU teams with iceboxes and icepacks for adequate transport of specimens at 4–8 °C. However, remote provinces noted consistent delays in specimen transport, which was exacerbated by the COVID-19 pandemic, the country’s ongoing economic crisis and political instability. The specimen’s referral from sites to the central level was organized on a weekly basis and integrated with specimens for testing for other communicable diseases and via transportation hubs located at the provincial level. The district authorities did not have the means to organize their own transport.

The timeliness of laboratory results has shown variability over the years. From 2014 to 2017, it reached the recommended target. Starting in 2018, the indicator fell significantly below the 80% target. This decline likely reflected shortages of test kits and an increased workload among laboratory staff without scaling up, in particular during the outbreak of 2018–2019, and during the COVID-19 pandemic. Indeed, the staff testing for measles was also involved in testing for COVID-19. In 2023, timeliness started to improve with the integration of laboratory results within the DHIS2 platform. However, the high turnover of lab staff has impacted the timeliness of results in 2024.

Several challenges affected the quality of suspected measles case investigations during the study period. Contextual factors such as concurrent public health emergencies like the COVID-19 pandemic, cholera outbreak and armed conflicts strained the overall surveillance system. Limitations in surveillance capacity included underreporting of outpatient cases, high staff turnover at both the ESU and health facilities levels, lack of access to annual vaccination coverage data at provincial and district levels, absence of a comprehensive childhood vaccination registry, and inadequate transportation logistics at the district level. Constraints in laboratory capacity further compounded these challenges, including insufficient human resources with no scale-up during outbreaks and staff turnover and shortages of essential laboratory reagents, particularly those needed for oral fluid testing.

## 5. Conclusions

During 2013–2024, measles surveillance performance, assessed against WHO-recommended indicators, met targets for timely case investigation and specimen adequacy. However, several key indicators fell below WHO targets, including surveillance sensitivity, completeness of vaccination data, and timeliness of specimen transportation and laboratory reporting.

Moreover, resource constraints and broader issues related to political and economic instability significantly hindered the progress toward measles elimination. To address these gaps, we recommend strengthening the reporting of suspected outpatient health facilities particularly from primary healthcare centers and private clinics. In addition, we recommend implementing community-based surveillance and continuous training for healthcare providers, ESU staff and community workers. Moreover, sustained investment in health information systems is crucial to link surveillance and vaccination databases. Finally, we recommend adequate procurement for the national measles laboratory designated for testing suspect cases.

## Figures and Tables

**Table 1:** List of measles surveillance performance indicators, Lebanon, 2013–2024.

Indicator	Numerator	Denominator	WHO Target
Reported rate of discarded measles cases	Number of notified measles cases tested negative for measles and rubella	Population/100,000 inhabitants	≥2/100,000
Timely investigation	Number of suspected measles cases timely investigated within 48 h of notification	Number of notified suspected measles cases	>80%
Adequate investigation	Number of suspected measles cases investigated including: age, sex, province, date of rash onset, date of investigation, vaccination status, date of last vaccination with measles-containing vaccine (MCV), date of specimen collection and travel history	Number of notified suspected measles cases	>80%
Testing for measles	Number of suspected measles cases tested for measles	Number of notified suspected measles cases	>80%
Testing with adequate conditions	Number of specimens collected within a defined period from rash onset *	Number of collected specimens	>80%
Timely transport of specimens to the laboratory	Number of specimens from measles suspected cases received at the laboratory within 5 days of collection	Number of specimens collected from suspected measles cases	>80%
Timely reporting of results by the laboratory	Number of specimens from suspected measles cases tested at the laboratory for which results were provided to ESU within 4 days of specimen receipt	Number of specimens collected from suspected measles cases	>80%

* The period depends on the type of test: For serology it is 28 days; for virus detection it is 14 days; for virus isolation it is 5 days.

**Table 2:** Evaluation of selected measles performance indicators by year of surveillance, Lebanon, 2013–2024.

Performance Indicator	Year of Surveillance												
	2013	2014	2015	2016	2017	2018	2019	2020	2021	2022	2023	2024	WHO Target
Discarded cases/100,000	4.2	1.8	0.7	1.1	1.3	3.8	5.5	0.2	0.1	1.1	6.2	2.3	≥2 cases/100,000 population
Cases investigated ≤48 h after notification	90	79	87	89	90	94	86	79	100	41	16	16	≥80%
Cases with adequate investigation	83	59	28	33	40	69	52	25	53	30	73	72	≥80%
Suspected cases tested for measles	64	65	58	79	69	72	77	36	28	52	79	69	≥80%
Specimens with adequate conditions	98	100	96	97	97	100	100	100	100	96	98	99	≥80%
Timeliness of specimens’ transport	28	32	44	70	59	19	3	0	0	22	59	23	≥80%
Timeliness of reporting laboratory results	44	92	100	98	83	25	15	10	60	44	74	19	≥80%

**Table 3:** Evaluation of selected measles performance indicators by province, Lebanon, 2013–2024.

Performance Indicator	Province								
	Beirut	Mount Lebanon	North	Akkar	South	Nabatieh	Bekaa	Baalbeck-Hermel	Target per WHO (%)
Discarded cases/100,000	1.2	0.9	2.8	3.4	1.7	2.5	2.8	10	≥2 cases/100,000 population
Cases investigated ≤48 h after notification	76	74	67	63	76	73	71	70	≥80%
Cases with adequate investigation	48	53	48	52	45	37	47	50	≥80%
Suspected cases tested for measles	71	61	64	62	70	78	62	66	≥80%
Specimens with adequate conditions	98	99	98	89	99	100	99	100	≥80%
Timeliness of specimens’ transport	44	44	19	27	46	27	31	17	≥80%
Timeliness of reporting laboratory results	56	59	56	50	63	58	55	52	≥80%

## Data Availability

The raw data supporting the conclusions of this article will be made available by the authors on request.
